# Cyanobacterial Circadian Clock: Molecular Mechanisms and Physiological Outputs

**DOI:** 10.3390/plants15152293

**Published:** 2026-07-27

**Authors:** Xiaobing Hu, Xin Ning, Jiewei Zhang, Dan Zhu

**Affiliations:** 1 School of Environmental Engineering, Yellow River Conservancy Technical University, Kaifeng 475004, China; 2008810228@yrcti.edu.cn; 2 Key Laboratory of Photobiology, Institute of Botany, Photosynthesis Research Center, Chinese Academy of Sciences, Beijing 100093, China; ningxin@henu.edu.cn; 3 Beijing Key Laboratory of Agricultural Genetic Resources and Biotechnology, Beijing Key Laboratory of Crop Molecular Design and Intelligent Breeding, Beijing Academy of Agriculture and Forestry Sciences, Beijing 100097, China

**Keywords:** cyanobacteria, circadian rhythms, core oscillator, Kai proteins, regulatory network

## Abstract

Earth’s rotation produces day and night cycles that are a primary selective pressure driving the evolution of endogenous circadian clocks. Cyanobacteria are the most studied prokaryotic model, and their timekeeping core is a protein oscillator composed of KaiA, KaiB, and KaiC. This oscillator sustains a near-24 h rhythm independently of transcription–translation feedback, challenging the long-standing assumption that prokaryotes merely respond passively to environmental cues. Moreover, it offers unique insights into the evolution and operational logic of circadian clocks. This review summarizes advances in cyanobacterial circadian research. We first analyze the KaiABC oscillator’s molecular basis, including synergistic conformational changes, phosphorylation and dephosphorylation cascades, and temperature compensation, which confer robustness and tunability. We compare oscillator compositions across cyanobacterial lineages, showing evolutionary plasticity. We then outline input and output networks, clarifying how environmental signals reset the oscillator phase and how temporal information is relayed to downstream processes. We further explain how the clock coordinates photosynthesis, nitrogen fixation, respiration, and cell division through predictive regulation, temporal decoupling, and resource prioritization, thereby resolving metabolic conflicts and enhancing fitness under light and dark cycles. This framework provides a theoretical basis for microbial survival strategies in fluctuating environments and offers insights for synthetic biology circuit design. Finally, we discuss open questions, including coupling between the oscillator and the cell cycle, functional divergence among ecotypes, and roles at the community level. Further research on the cyanobacterial clock will help clarify general principles of biological timing and its evolutionary origins.

## 1. Introduction

The Earth’s rotation gives rise to periodic environmental fluctuations such as diurnal light–dark cycles and temperature variations. To adapt to these regular environmental challenges, life on Earth has broadly evolved endogenous circadian timing systems, known as circadian clocks [1]. This highly conserved system enables organisms to anticipate and pre-adjust their physiology, metabolism, and behavior to synchronize with environmental cycles, thereby conferring significant adaptive advantages [2].

Circadian clocks exhibit both deep universality and remarkable diversity across life forms. From structurally simple prokaryotes to higher eukaryotes, functionally distinct rhythmic systems have emerged through evolution [3,4]. In eukaryotes, complex clock networks in animals and fungi have been extensively characterized [5]. In contrast, whether prokaryotes possess autonomous endogenous clocks remained controversial for decades. Definitive evidence emerged only upon the discovery of self-sustained, free-running rhythms of approximately 24 h in cyanobacteria that persist independently of external cues [6]. Cyanobacteria are a phylogenetically distinct phylum of oxygenic photosynthetic prokaryotes that inhabit diverse aquatic and terrestrial ecosystems. As the only prokaryotes capable of plant-like oxygenic photosynthesis, they play pivotal roles in global carbon and nitrogen cycles. Rather than being merely a tractable model for dissecting clock mechanics, their prokaryotic organization provides a critical window into the origin and early evolution of circadian systems [7].

Landmark advances in cyanobacterial chronobiology began in the 1980s. At that time, researchers observed temporal partitioning of nitrogen fixation and photosynthesis in unicellular diazotrophic cyanobacteria. This implied the presence of an intrinsic timing mechanism. Subsequent studies in the model strain *Synechococcus elongatus* PCC 7942 identified the core oscillator comprising three essential proteins: KaiA, KaiB, and KaiC [8,9]. In 2005, a groundbreaking study successfully reconstituted sustained, temperature-compensated oscillations at the post-translational level in vitro using only these three proteins and ATP [10]. This pioneering work established the cyanobacterial clock as a “protein-based clock” and laid the biochemical foundation for dissecting the core oscillator [11]. Beyond this central pacemaker, input pathways relay environmental signals to the core, while output pathways transmit temporal information to downstream physiological processes, thereby orchestrating key functions including photosynthesis, cell division, metabolism, and population behavior, which collectively confer robust competitive fitness [12].

Although the molecular framework of the cyanobacterial clock is largely established in model strains such as *S. elongatus* PCC 7942, several frontiers remain to be explored. These include the diversity of core oscillators among ecologically distinct cyanobacterial lineages and their adaptive significance, the fine-tuning roles of post-translational modifications, how the clock coordinates metabolic network dynamics, and the potential engineering of novel timing modules for synthetic biology applications. Accordingly, this review first provides a systematic overview of the molecular mechanisms and biochemical basis of the cyanobacterial core oscillator, followed by a synthesis of the regulatory networks governing input and output pathways. We then elucidate how this rhythmic system integrates and drives key physiological and ecological processes. Finally, we highlight future challenges and research directions in the field, aiming to offer a comprehensive perspective on the general principles and evolutionary origins of circadian clocks.

## 2. The Core Oscillator of the Cyanobacterial Circadian Clock: A Prokaryotic Protein-Based Chemical Oscillator

Cyanobacteria represent the most thoroughly characterized prokaryotes confirmed to possess canonical endogenous circadian rhythms [13]. Elucidation of their clock system has fundamentally challenged the traditional notion that “simple prokaryotic structures preclude the need for sophisticated endogenous timing” and has established them as a pivotal model for exploring the origin and core mechanisms of biological clocks [14,15]. Among them, *S. elongatus* PCC 7942 serves as the primary model system for studying prokaryotic clocks due to its genetic tractability and robust rhythmicity. This system exhibits hallmark features characteristic of eukaryotic clocks, including free-running periodicity, entrainment to environmental cues, and temperature compensation [16]. More notably, it operates via a distinctive “protein-based timing oscillator” that functions independently of transcription–translation feedback loops (TTFLs), thereby redefining the theoretical framework for understanding the generation of biological rhythms [17].

### 2.1. KaiC: The Timing Core and Phosphorylation Engine

KaiC acts as the structural and catalytic hub of the oscillator, functioning as a homohexamer. Each monomer comprises two domains: the N-terminal CI domain and the C-terminal CII domain, which stack vertically to form a double-ring structure [18]. The CI ring possesses ATPase activity, providing energy for the oscillation, whereas the CII ring harbors two key phosphorylation sites: threonine 432 (T432) and serine 431 (S431). The ordered phosphorylation and dephosphorylation cycle at these sites constitutes the core “pendulum” driving the near-24 h period. Phosphorylation follows a strict temporal sequence: T432 is phosphorylated first, followed by S431, establishing a doubly phosphorylated state (pST). Dephosphorylation subsequently proceeds in the same order (T432 first, then S431) to reset the system [19]. Throughout this process, changes in phosphorylation status precisely regulate the conformation of KaiC, thereby dictating its affinity for KaiA and KaiB, thus forming the structural basis of timing.

### 2.2. KaiA: The Positive Regulator of Phosphorylation

KaiA functions as a positive regulator, typically acting as a dimer to promote KaiC phosphorylation and drive the oscillator into the “daytime” (phosphorylation accumulation) phase [20]. Each KaiA monomer contains a four-helix bundle domain, extending into a highly flexible, arginine-rich C-terminal “tail loop”. It is via this tail loop that KaiA specifically binds to the C-terminal flexible peptide of low-phosphorylated KaiC hexamers [21]. This interaction stabilizes the active conformation of KaiC, dramatically enhancing its autokinase activity and accelerating the phosphorylation of T432 and S431. At the initiation stage of the oscillation, KaiA binding serves as the key “winding” mechanism to start the phosphorylation cycle.

### 2.3. KaiB: Phase Transition and the Captor of KaiA

KaiB serves as the crucial negative regulator, responsible for initiating the phase transition from “day” to “night” [22]. KaiB adopts a thioredoxin fold, but its functional activation depends on a critical phase transition event. KaiB exists in a dynamic equilibrium between a ground-state dimer and a functionally competent, fold-switched tetramer; the tetrameric form is sparsely populated in the absence of KaiC. As KaiC approaches full phosphorylation (pST state), KaiB monomers undergo a conformational shift to assemble into tetramers. This allows them to specifically bind to the conformationally rearranged KaiC-pST, forming the KaiB–KaiC complex [23]. This complex acts as an efficient trap capable of capturing free KaiA. The binding of KaiB to KaiA competitively blocks KaiA’s interaction with KaiC, thereby relieving KaiA’s stimulatory effect [24]. Concurrently, the intrinsic phosphatase activity of KaiC dominates, leading the system into the dephosphorylation phase and completing the phase switch [25].

### 2.4. The Core Oscillation Cycle: Precise Dynamics of the Three-Protein Reaction Network

**Figure 1:**
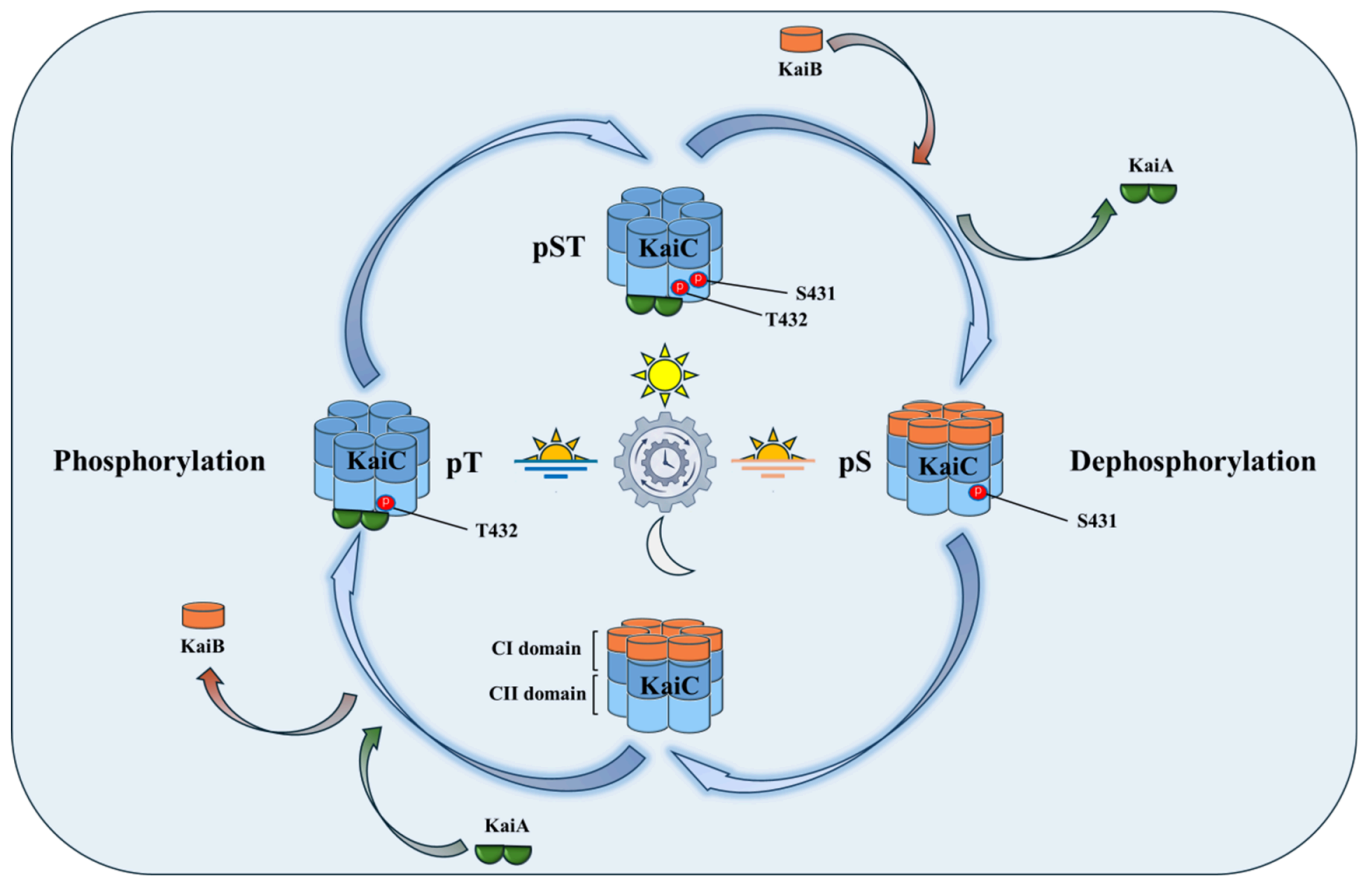
The KaiA–KaiB–KaiC ternary protein phosphorylation–dephosphorylation cycle underlying the cyanobacterial core oscillator.

The autonomous oscillation of the cyanobacterial core clock arises from the interplay among KaiA, KaiB, and KaiC and the coupling of KaiC phosphorylation status, together constituting a complete phosphorylation–dephosphorylation cycle [26]. Importantly, both kinase and phosphatase activities are intrinsic to KaiC: KaiA stimulates KaiC’s autokinase activity, whereas dephosphorylation is catalyzed by KaiC itself without the requirement for exogenous enzymes. During the daytime phase, dephosphorylated KaiC binds KaiA. This stimulates KaiC’s autokinase activity, leading to sequential phosphorylation at residues T432 and S431 and a progressive elevation in the overall phosphorylation level of the system. As dusk approaches, hyperphosphorylated KaiC (pST) undergoes a global conformational transition that exposes the KaiB-binding interface. KaiB then undergoes a phase transition and engages KaiC, forming the KaiB–KaiC complex. Throughout the night, the KaiB–KaiC complex sequesters and inactivates KaiA, allowing the phosphatase activity of KaiC to dominate, which drives the stepwise removal of phosphate groups from T432 and subsequently S431, thereby reducing the system’s phosphorylation level. Prior to dawn, fully dephosphorylated KaiC reverts to its initial conformation and releases KaiA and KaiB. Freed KaiA rebinds KaiC, initiating a new round of the oscillatory cycle (Figure 1). The elegance of this cycle lies in its chemical self-sufficiency: driven entirely by protein–protein interactions, conformational dynamics, and post-translational modifications without requiring transcription or translation, it represents the simplest known autonomous circadian oscillator [27].

### 2.5. Complexity Case Study: The Oscillator Network of Synechocystis sp. PCC 6803

The circadian system in *Synechocystis* sp. PCC 6803 exhibits considerably greater complexity than that of *S. elongatus* PCC 7942, characterized by the networking and functional diversification of its core oscillator components [28]. Its genome encodes multiple Kai protein paralogs, specifically two KaiA proteins (KaiA1 and KaiA3), three KaiB proteins (KaiB1–B3), and three KaiC proteins (KaiC1–C3) [29]. The KaiAB1C1 system most closely resembles the canonical oscillator of *S. elongatus* PCC 7942, functioning as the primary circadian pacemaker capable of coupling to downstream signaling cascades such as the SasA–RpaA pathway to drive genome-wide transcriptional rhythms [30]. In contrast, the KaiB2C2 module plays a unique role. Although disruption of *kaiB2C2* leaves circadian rhythms intact, *kaiC2* is indispensable for viability and cannot be fully deleted. This suggests that KaiB2C2 is not a direct component of the core clock, but instead underpins fundamental cellular processes such as basal metabolism and the cell cycle. Further complexity arises from the KaiA3B3C3 system, which forms a functional phosphorylation cycle in vitro [31]. Deletion of this module destabilizes the period and impairs growth under metabolic switching conditions, indicating its role in fine-tuning clock outputs to optimize energy allocation [32,33]. Collectively, these findings suggest that the *Synechocystis* clock comprises a network of partially divergent yet coupled “sub-oscillators”. This architecture provides enhanced robustness against environmental perturbations and positions this strain as a unique model for dissecting the evolution of circadian networks [34,35].

### 2.6. Diversity and Evolutionary Implications of Cyanobacterial Core Oscillators

Genomic analyses have revealed remarkable evolutionary diversity in the core components of the cyanobacterial circadian clock, challenging the prevailing notion that the KaiABC trio constitutes the sole canonical oscillator [36]. The distribution of *kaiA*, *kaiB*, and *kaiC* varies drastically across the phylum, reflecting extensive genomic streamlining and functional divergence.

In contrast to the canonical KaiABC oscillator in *S. elongatus* PCC 7942, marine *Prochlorococcus* strains (e.g., MIT9313, MED4) exhibit a streamlined genome lacking *kaiA* while retaining only *kaiB* and *kaiC*. Rhythm generation in these organisms may stem from the intrinsic slow oscillation of KaiC, compensation by unidentified proteins, or tight coupling to metabolic cues such as redox states or ATP/ADP ratios. This indicates that KaiA is not an absolute prerequisite for autonomous timekeeping [37]. Further reductions are observed in other lineages. Notably, in filamentous Nostocales, KaiA displays marked N-terminal truncation (~100–200 aa), resulting in the loss of part of the amplitude-amplifier domain while preserving the C-terminal region essential for oscillation. In the endosymbiont *Candidatus Atelocyanobacterium thalassa* (UCYN-A) and certain *Synechococcus* isolates, *kaiB* is completely absent despite *kaiC* retention, suggesting that a KaiC-only timer suffices in nutrient-poor or symbiotic niches [38]. Similarly, the basal unicellular lineage *Gloeobacter violaceus* PCC 7421 lacks thylakoid membranes and all canonical clock components (*kaiABC*) along with major output factors (e.g., *sasA*, *cikA*) [39]. This minimal configuration implies either the existence of highly divergent Kai-like proteins undetectable by conventional homology searches or the adoption of entirely distinct timing mechanisms. Collectively, these manifestations—encompassing total gene loss, partial truncations, and domain simplification—demonstrate that the cyanobacterial clock is not absolutely conserved. Rather, it constitutes a modular repertoire of functional genes shaped by ecological specialization and genomic economy.

Extending beyond Cyanobacteria, homologs of *kaiB* and *kaiC* are sporadically distributed across diverse bacterial phyla (e.g., *Rhodobacter sphaeroides* in Proteobacteria, *Chloroflexi*, and *Bacteroidetes*) and even in Archaea (e.g., *Pyrococcus* and *Thermococcus*), whereas KaiA remains essentially exclusive to this phylum [40,41]. Although non-cyanobacterial KaiC invariably retains autokinase activity, it functions without the sophisticated KaiABC interaction network, likely driving simple diurnal oscillations or serving non-clock roles in stress response and metabolism. These investigations elucidate the physicochemical underpinnings of circadian timing and, critically, illuminate the origins and environmental co-adaptation of biological clocks [42].

## 3. The Regulatory Network of the Cyanobacterial Clock: Systemic Integration of Input, Core, and Output

As an autonomous protein-based chemical oscillator, the cyanobacterial circadian clock relies on the precise perception of environmental cues, fine-tuning of the core oscillator phase, and rhythmic output to downstream physiological processes. These three elements constitute a hierarchically organized and intricately regulated network.

### 3.1. Input Pathways: Indirect Sensing via Metabolic Signals

For a circadian clock to function effectively, it must maintain synchrony with environmental time cues, most notably the diurnal light–dark cycle [43]. In cyanobacteria, entrainment is achieved not through dedicated photoreceptors directly coupled to the core oscillator—such as the phytochromes found in plants—but primarily via metabolic consequences of photosynthesis [44]. Light-driven photosynthetic activity remodels key intracellular metabolic parameters, including the ATP/ADP ratio, the NADPH/NADP^+^ balance, and the redox state of the plastoquinone pool [45]. These metabolic shifts feed into the core oscillator, modulating KaiC ATPase activity or the kinetics of its phosphorylation cycle to reset the phase of the clock [46]. Central to this process is the histidine kinase CikA, which senses cellular redox fluctuations and relays this information to the Kai oscillator, thereby aligning the internal timekeeping mechanism with external light–dark cycles [47]. Temperature cycles can also serve as entraining cues; although the Kai oscillator exhibits temperature compensation, pronounced diel temperature fluctuations still influence clock pace [48]. The underlying mechanisms likely involve temperature-dependent alterations in protein folding, interaction dynamics, or metabolic flux, though the precise molecular details remain to be fully elucidated [49].

### 3.2. Post-Translational Regulation and Transcriptional Feedback of the Core Oscillator

The KaiA, KaiB, and KaiC proteins constitute an autonomous circadian pacemaker through their phosphorylation cycle and dynamic interactions, a core mechanism that is further reinforced by transcriptional feedback loops [50]. As detailed in Section 2.4, the phosphorylation status of KaiC—specifically the mono-phosphorylated (pS, pT) and doubly phosphorylated (pST) states—periodically modulates its affinity for KaiA and KaiB, driving the assembly and disassembly of transient complexes. While KaiA stimulates KaiC autophosphorylation, KaiB acts at a specific phase to sequester and inhibit KaiA, thereby initiating the dephosphorylation phase. Powered by ATP hydrolysis, the intrinsically slow kinetics of this cycle establish the biophysical basis for the near-24 h period [51]. Although this core oscillation is independent of transcription, it is tightly coupled within the cell to a transcription–translation feedback loop that serves to amplify and stabilize the rhythm. Phosphorylated KaiC, particularly the pST form, represses transcription of its own *kaiBC* operon. As KaiC dephosphorylates, this repression is relieved, resulting in rhythmic accumulation of *kaiBC* mRNA and protein that peaks out of phase with the core phosphorylation cycle. This cyclical fluctuation in protein abundance superimposes upon the post-translational oscillator, markedly enhancing the amplitude of output signals and bolstering the overall robustness of the system [52].

### 3.3. Output Pathways: From Core Oscillation to Global Physiological Rhythms

Rhythmic signals generated by the core oscillator are transmitted genome-wide primarily through two major signaling pathways, regulating the circadian expression of downstream genes.

#### 3.3.1. The SasA-RpaA Two-Component System

This is the predominant output route. The histidine kinase SasA directly binds to hyperphosphorylated KaiC and becomes activated. Activated SasA autophosphorylates and subsequently transfers the phosphate group to an aspartate residue on the response regulator RpaA [53]. Phosphorylated RpaA (RpaA~P) dimerizes, enhancing its DNA-binding capability to activate or repress the transcription of a large repertoire of target genes involved in photosynthesis, metabolism, and nitrogen fixation. When KaiC dephosphorylates, SasA activity declines, and RpaA~P is inactivated via autodephosphorylation or phosphatase action, terminating the signal. Knockout of *sasA* or *rpaA* abolishes the rhythmicity of the vast majority of gene expression. Although the core KaiC phosphorylation rhythm persists, the clock can no longer drive physiological changes, rendering it a “dumb clock” [54].

#### 3.3.2. The CikA Pathway

CikA functions as a critical auxiliary regulator primarily responsible for signal tuning and homeostasis maintenance. Specific KaiC states (via KaiB) recruit CikA, which acts as a phosphatase to dephosphorylate RpaA~P, thereby negatively modulating the output strength of the SasA–RpaA pathway. Additionally, CikA itself perceives environmental cues such as redox status. Consequently, the CikA circuit functions akin to a “dimmer switch” and “environmental responder”; it does not determine the phase of the rhythm but fine-tunes the amplitude and duration of output signals based on cellular energy status and environmental conditions, thereby enhancing the robustness and adaptability of the circadian clock [55].

### 3.4. Coordination of Input, Core, and Output Modules

**Figure 2:**
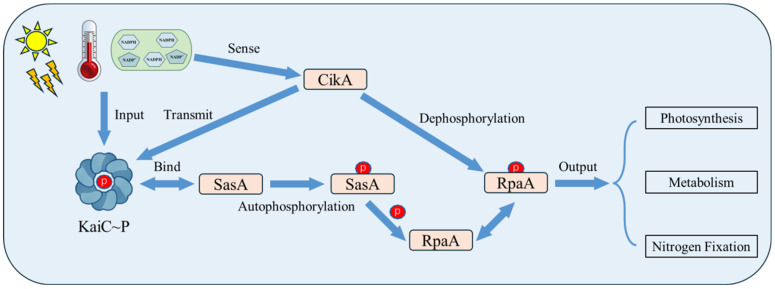
Hierarchical regulatory network of the cyanobacterial circadian clock: systemic integration of input, core, and output modules.

The cyanobacterial circadian regulatory network constitutes a multi-tiered integrative system. The input tier transduces environmental signals—primarily light-derived metabolic cues—into chemical signals that are legible to the core oscillator. The core tier generates autonomous, robust timing signals through the post-translational interactions and phosphorylation cycle of the Kai proteins. The output tier broadcasts these timing signals across the genome via the primary SasA–RpaA cascade, while auxiliary circuits such as CikA provide dynamic modulation [56]. This exquisite architectural design enables cyanobacteria to coordinate their physiological activities with exceptional spatiotemporal precision, ensuring optimal adaptation to the cyclic day–night environment (Figure 2) [57].

## 4. Biological Functions of the Cyanobacterial Clock: Spatiotemporal Coordination of Core Physiological Processes

The cyanobacterial circadian clock is far more than a simple gene expression oscillator; it functions as an integrative “central dispatch system” [58]. Its core mandate lies in prediction, coordination, and optimization. Through an intrinsic near-24 h rhythmic program, it strategically regulates pivotal life processes—including photosynthesis, nitrogen fixation, respiration, and cell division—precisely decoupling or coupling them in time and space [59]. This maximizes energy efficiency, circumvents inherent metabolic conflicts, and enhances adaptability and resilience to environmental fluctuations [60].

### 4.1. Photosynthesis: Integrative Strategies of Prediction, Optimization, and Protection

The integration of the circadian clock with photosynthesis represents a defining exemplar of chronobiology in photoautotrophs, wherein the clock orchestrates a continuum of anticipatory and protective measures to maximize efficiency and resilience [61]. Leveraging its predictive capacity, the clock drives anticipatory upregulation of photosystem subunits, electron transport chain components, and Calvin cycle enzymes just prior to dawn, ensuring the photosynthetic apparatus is fully primed upon light onset. Extending beyond diurnal anticipation, the clock functions as a photoperiod counter, sensing seasonal variations in day length to initiate cold-responsive gene expression and membrane lipid remodeling under short-day regimes, thereby enhancing low-temperature tolerance [62]. To maintain efficiency, the clock dynamically modulates light-harvesting antenna size in register with diurnal irradiance fluctuations, averting excess excitation energy and light limitation [63]. Crucially, the clock reconciles oxygenic photosynthesis and nitrogen fixation through temporal partitioning in unicellular cyanobacteria: oxygenic photosynthesis is confined to daylight, while nitrogen fixation occurs at night within the same cellular compartment but under micro-oxic conditions generated by respiratory O_2_ consumption. Concurrently, the clock orchestrates glycogen metabolism, channeling daytime carbon reserves into nighttime catabolism to fuel nitrogen fixation [64,65]. Confronted with high-light stress, the clock upregulates, prior to dawn, the expression of the PSII core protein D1 (*psbA*) and repair proteases such as FtsH, enabling rapid PSII restoration following photodamage [66].

### 4.2. Nitrogen Fixation: Temporal Partitioning and Alternative Strategies

Nitrogen fixation in unicellular cyanobacteria such as *S. elongatus* PCC 7942 illustrates the resolution of metabolic incompatibility through temporal partitioning. Oxygenic photosynthesis and nitrogenase activity are separated across the diel cycle: photosynthesis occurs during the light period, whereas nitrogen fixation is confined to the dark, when residual O_2_ is actively scavenged by respiration, creating a reduced microenvironment suitable for nitrogenase. This temporal separation is complemented by nocturnal glycogen catabolism, which supplies the 16–24 ATP molecules required to reduce each N_2_ molecule [14]. However, temporal partitioning is not universal. Filamentous heterocystous cyanobacteria (e.g., *Nostoc flagelliforme*) employ spatial segregation, differentiating thick-walled heterocysts that restrict O_2_ diffusion and maintain a micro-oxic interior for nitrogenase [67]. Conversely, colonial cyanobacteria such as *Trichodesmium* spp. fix nitrogen during the daytime, relying on rapid respiratory O_2_ consumption and spatial heterogeneity within trichomes to protect nitrogenase [68]. These diverse strategies highlight the evolutionary flexibility of cyanobacterial oxygen management mechanisms and caution against generalizing findings from any single model species [69].

### 4.3. Respiration: Central Hub for Diurnal Energy Homeostasis and Metabolic Interfacing

Under circadian governance, respiration transcends its basal role as an ATP generator to fulfill three interrelated functions essential for cellular coherence. First, the clock suppresses specific respiratory components during the day. It targets the cyanide-insensitive oxidase (Cyd) to prevent futile cycling. This minimizes competition with photosynthetic electron transport. Consequently, the cell maximizes the conversion of light energy into glycogen storage [70]. Second, with the onset of darkness, respiration transitions to the primary metabolic engine. The clock drives the anticipatory upregulation of genes involved in glycogenolysis and the respiratory chain, ensuring the immediate oxidation of glycogen reserves to sustain ATP supply. This nocturnal respiratory surge is particularly critical for powering energy-intensive processes such as nitrogen fixation and amino acid biosynthesis [71]. Third, respiration acts as a guardian of the cellular microenvironment. By upregulating high-affinity terminal oxidases, such as the cytochrome bdquinol oxidase, the clock actively depletes residual oxygen at night. This creates the strict micro-oxic conditions required to protect the oxygen-labile nitrogenase enzyme [72]. Reinforcing this coordination, the clock rhythmically upregulates antioxidant enzymes—including superoxide dismutase and catalase—timing their peaks to coincide with elevated respiratory activity. This synchronization efficiently scavenges reactive oxygen species (ROS) generated during electron transport, thereby shielding the cell from oxidative damage.

### 4.4. Cell Division: Strategic Gating of a High-Risk Cellular Event

The clock imposes stringent temporal control over cell division, restricting this vulnerable and energetically costly process to the safest and most propitious window, typically spanning the late night to predawn hours, so as to minimize risk and maximize replicative fidelity [73]. The clock schedules cell division outside periods of intense solar irradiation. This shields exposed chromosomal DNA from UV-induced damage. It also circumvents oxidative pressure from daytime photosynthesis. Simultaneously, it avoids competition for resources with high-intensity carbon fixation [74]. This predawn division window aligns seamlessly with the waning phase of nighttime respiratory energy provision, ensuring that the substantial ATP demands of chromosome replication and cytokinesis are adequately met [75]. Through the rhythmic modulation of key cell division proteins, such as the FtsZ ring component, and potentially through influences on factors governing the initiation of DNA replication, the clock establishes a stringent temporal gate that ensures cell division is executed in precise coordination with both intracellular metabolic status and extrinsic environmental conditions [76].

### 4.5. Integrated Physiological Network: A Synthesis

**Figure 3:**
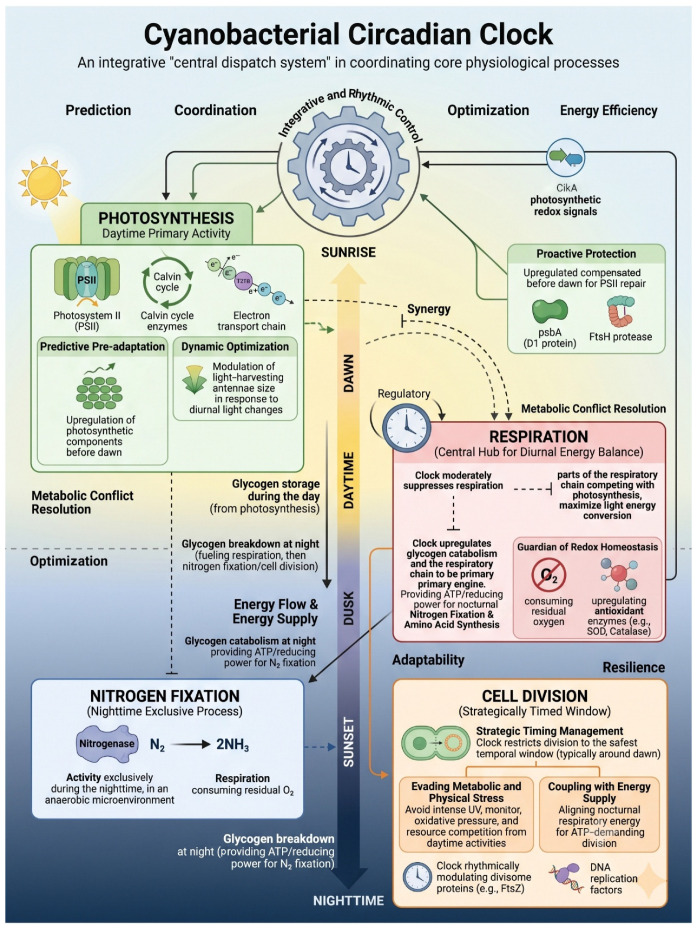
Spatiotemporal coordination of core physiological processes by the cyanobacterial circadian clock.

The cyanobacterial circadian clock weaves photosynthesis, nitrogen fixation, respiration, and cell division into a coherent, temporally ordered, functionally synergistic, and bioenergetically optimized adaptive network. By precisely scheduling these processes, the clock resolves intrinsic metabolic incompatibilities while simultaneously enabling the cell to anticipate environmental shifts and allocate resources with maximal efficiency [77]. This confers a decisive competitive advantage within the rigorous yet predictable diurnal landscape. Investigations into the functional repertoire of the cyanobacterial clock thus provide a paradigmatic framework for understanding the central role of biological rhythms in driving fundamental life processes (Figure 3) [78].

## 5. Discussion and Future Perspectives

Research on cyanobacteria has fundamentally reshaped our understanding of the origin and molecular foundations of biological clocks. The discovery and mechanistic dissection of the KaiABC core oscillator revealed that a post-translational, protein-based biochemical oscillator—operating independently of transcription–translation feedback loops—is sufficient to generate a robust, endogenous near-24 h rhythm. This paradigm-shifting model established the physicochemical feasibility of protein-based timekeeping and provided pivotal insights into the evolutionary precursors of more complex eukaryotic clocks.

Looking forward, several unresolved issues merit critical attention. First, a central question is why cyanobacterial lineages exhibit such striking diversity in Kai system architecture. We propose that this diversity reflects adaptation to environmental predictability. In shallow freshwater mats, diel cycles are highly predictable. Here, the canonical KaiABC oscillator provides high-amplitude, precise rhythms. In oligotrophic open oceans, *Prochlorococcus* experiences weaker selective pressure for precision, favoring streamlining to a KaiC-only oscillator coupled to metabolism. Filamentous nitrogen-fixers such as *Nostoc flagelliforme* integrate KaiABC with developmental programs, suggesting that oscillator complexity scales with physiological integration requirements. We term this continuum “ecological chronotypes”. Second, whether KaiC alone sustains robust rhythms remains debated. In vitro, KaiC oscillates without KaiA under specific ATP/ADP ratios, confirming its intrinsic timing potential. In vivo, however, the requirement for KaiA varies among lineages. In *S. elongatus*, kaiA deletion abolishes rhythmicity, whereas kaiA-null strains of *Prochlorococcus* retain genome-wide transcriptional rhythms. These discrepancies may reflect metabolic compensation for the loss of KaiA-mediated phosphorylation. Alternatively, functional redundancy by uncharacterized regulators may account for this variability. Finally, from a translational perspective, the modular design of the KaiABC system presents an attractive platform for engineering biology. Reconstructing or engineering novel oscillators within synthetic circuits may inspire applications ranging from timed drug delivery to precision bioproduction [79].

Continued investigation into the cyanobacterial circadian clock promises not only to deepen our comprehension of fundamental timing mechanisms but also to unveil the universal logic governing temporal regulation across life forms. As a minimal yet fully functional model, the cyanobacterial system will remain an indispensable paradigm for exploring the spatiotemporal strategies that enable life to anticipate, adapt, and thrive within a rhythmic environment.

## Data Availability

No new data were created or analyzed in this study. Data sharing is not applicable to this article.
